# Trimetazidine Ameliorates Myocardial Metabolic Remodeling in Isoproterenol-Induced Rats Through Regulating Ketone Body Metabolism *via* Activating AMPK and PPAR α

**DOI:** 10.3389/fphar.2020.01255

**Published:** 2020-08-14

**Authors:** Huihui Li, Zhi Ma, Yajun Zhai, Chao Lv, Peng Yuan, Feng Zhu, Liping Wei, Qi Li, Xin Qi

**Affiliations:** ^1^School of Graduate Studies, Tianjin University of Traditional Chinese Medicine, Tianjin, China; ^2^Department of Cardiology, Tianjin Union Medical Center, Tianjin, China; ^3^Graduate School, Tianjin Medical University, Tianjin, China

**Keywords:** trimetazidine, myocardial metabolic remodeling, heart failure, ketone body metabolism, AMPK, PPARα

## Abstract

**Background:**

Metabolic remodeling plays a vital role in the development of heart failure. The trimetazidine can optimize fatty acid and glucose oxidation *via* inhibition of long-chain 3-ketoacyl CoA thiolase in the heart. So, trimetazidine commonly used in cardiovascular therapy as a myocardial metabolic drug. This study was conducted to assess the effects and mechanisms of trimetazidine on ketone body metabolism in heart failure rats.

**Methods:**

A rat model of heart failure was established by continuous subcutaneous injection of isoproterenol in 10 mg/kg/d. We examined body weight, heart weight index, and tested B-type natriuretic peptide by kit. We detected the structure and function of the heart. Hematoxylin-eosin staining and Masson’s trichrome staining was performed to assess myocardial tissue morphology. To evaluate apoptosis, we used Tunel staining. Metabolic substrate contents of glucose, free fatty acid, ketone bodies, lactic acid, and pyruvate and ATP levels in myocardial tissues were measured with the corresponding kit. We detected the levels of protein expressions related to myocardial substrate uptake and utilization by Western blot.

**Results:**

Trimetazidine remarkably reduced the heart weight index and B-type natriuretic peptide levels. Besides, trimetazidine increased the level of blood pressure and decreased heart rate. Moreover, trimetazidine inhibited decreases in left ventricular ejection fraction and left ventricular fractional shortening. Further, trimetazidine decreased the levels of collagen volume fraction and promoted ATP production in myocardial tissues. Trimetazidine also reduced the levels of free fatty acid, ketone bodies, lactic acid, and increased glucose and pyruvate levels in myocardial tissues. Furthermore, trimetazidine markedly inhibited apoptosis. More importantly, the protein expression levels related to myocardial substrate uptake and utilization increased dramatically in the trimetazidine group. In particular, the protein expressions related to ketone body utilization were prominent.

**Conclusions:**

Trimetazidine could attenuate metabolic remodeling and improve cardiac function in heart failure rats. The potential mechanism for the cardioprotective effect of trimetazidine may be highly associated with its regulation of adenosine monophosphate-activated protein kinase, and peroxisome proliferator activated receptor α expressions. Along with the regulation, myocardial substrate utilization was improved, especially the utilization of ketone bodies.

## Introduction

Heart failure (HF), with its notable morbidity and mortality, is directly related to significant health and economic burdens worldwide (Benjamin et al., 2017). The current therapies to treat HF primarily address the neurohumoral systems, including the renin-angiotensin-aldosterone system and the β- adrenergic receptor signaling pathway (Ponikowski et al., 2016). Increasing evidence suggests that the development of heart failure accompanied derangements in myocardial energy metabolism (Rosano and Vitale, 2018).

Metabolic failure is considered as a vital role in the HF pathological process. Evidence grows that myocardial energy metabolism in patients with HF shows a confusing picture, leading to the development and deterioration of the disease (Birkenfeld et al., 2011). The heart, a high metabolic rate organ, needs to regulate the substrate supply to satisfy various states (Karwi et al., 2018). Some substrates can be used for energy metabolism in the heart, including free fatty acids (FFA), carbohydrates (glucose and lactate), ketone bodies, and amino acid (Kolwicz et al., 2016). In the mature heart, adenosine triphosphate (ATP) production relies mostly on fatty acid oxidation while carbohydrates and ketone body (KB) provide part of ATP generation. Along with reducing ATP production, the heart is considered to be energy deficient in the progression of heart failure (Chen et al., 2019). The failing heart could improve ATP production by reducing cardiac fatty acids utilization and an increase in glucose use. Cardiomyocytes can use many other substrates, including ketone bodies, lactate, and amino acids, except glucose and fatty acids. More recently, growing evidence indicates that ketone bodies compete with other substrates used for fuel in the failing heart (Bedi et al., 2016). High competition of ketone body leads to an increase in ketone production and cardiac ketone utilization (Nakamura and Sadoshima, 2019). Although the ketone body utilization behaves as a potential benefit, few previous studies have revealed its usage related to the energy metabolism in HF. Therefore, further research should be conducted about ketone body utilization mechanisms in the pathological process of heart failure.

Trimetazidine (TMZ) is the most investigated drug in energy metabolism. TMZ can shift the utilization of metabolic substrate from fatty acid to glucose to satisfy the energy demand (Wang et al., 2016). Recognizing this, TMZ has the potential to be used to treat the metabolic remodeling of HF. Studies proved that TMZ could partially inhibit myocardial fatty acid oxidation. TMZ exerts its functions primarily through inhibition of the long-chain activity of the enzyme acetyl CoA C-acyltransferase, also known as 3-KAT. 3-KAT enzyme catalyzed the terminal reaction of fatty acid beta-oxidation. The reaction uses long-chain 3-ketoacyl CoA as a fuel to generate acetyl CoA (Lopaschuk et al., 2003). Studies show that 3-KAT in the mitochondrial matrix inhibits pyruvate dehydrogenase, while TMZ could eliminate this limitation *via* inhibiting 3-KAT (Li et al., 2019). TMZ significantly enhances the rate of glucose oxidation, modestly lowering the efficiency of fatty acid oxidation that has been confirmed.

Since ketone bodies can be generated by fatty acid, and TMZ could regulate a fatty acid metabolism, then whether TMZ acts on ketone body metabolism. If it works, how does TMZ affect the production and utilization of ketone body. AMPK, an energy sensor, functions as a vital regulator of metabolic homeostasis. Further, AMPK can protect myocardium from severe injury and alleviate energy exhaustion during heart failure development (Li et al., 2019). PPARα is a vital factor in the metabolic system, where it participates in signaling driven by AMPK (Grabacka et al., 2016). We try to explore TMZ regulating ketone body metabolism *via* AMPK and PPARα pathway in this study.

## Materials and Methods

### Reagents and Materials

We purchased isoproterenol (ISO) from Beijing Solarbio Science & Technology Co., Ltd. (Beijing, China) and obtained trimetazidine from Servier (Tianjin) Pharmaceutical Co., Ltd. (Tianjin, China) (Batch number: 2014432). We obtained primary antibodies against adenosine monophosphate-activated protein kinase (AMPK), peroxisome proliferator activated receptorα (PPARα), cleaved caspase 3, caspase 3, and cytochrome C from Wanlei bio (Shenyang, China). Also, primary antibodies against lactate dehydrogenase (LDH), β-actin, and glucose transporter 4 (GLUT4) were included. We obtained primary antibody against phospho-acetyl-CoA carboxylase (p-ACC) from Biyuntian Bio-Technology Co., Ltd. (Shanghai, China). The primary antibody against 3-hydroxy-3-methylglutaryl-CoA2 (HMGCS2) was obtained from Wuhan ABclonal Biotechnology Co., Ltd. (Wuhan, China). Primary antibody against monocarboxylate transporter 1 (MCT1), β-hydroxybutyrate dehydrogenase 1 (BDH1), and phospho-AMPK (p-AMPK) were obtained from Beijing Biosynthesis Biotechnology Co., Ltd. (Beijing, China). Primary antibody against carnitine palmitoyltransferase 1 (CPT1) and acetoacetyl-CoA thiolase 1 (ACAT1) were purchased from Abcam (Cambridge, UK). Primary antibody against succinyl-CoA:3-ketoacid-CoA transferase1(OXCT1) was purchased from Proteintech Group, Inc. (Wuhan, China). We bought the horseradish peroxidase (HRP)-tagged secondary antibody from Zsbio Biology (Beijing, China) and primary antibody against Anti-ATP1A1 from Wuhan Boster Biotechnology Co., Ltd. (Wuhan, China).

### Animal Model and Treatment Protocols

Our animal experiments were approved by the Animal Care and Use Committee of the Tianjin Union Medical Center. We used 36 male Sprague Dawley rats provided by the Vital River Laboratory Animal Technology Co., Ltd. (Beijing, China) for this study, which were weighted about 300–350 g. Rats were fed for one week in room temperature adaptability conditions, randomly allocated into standard control group (n = 10) and ISO-induced HF group (n = 26). All rats were raised in a standard environment with conventional laboratory food and water. The control group was given an injection of standard saline corresponding to the ISO-induced HF group. Meanwhile, the ISO-induced HF group was given a subcutaneous injection of ISO (Purity 98%) at 10 mg/kg/d. The infusion was consecutive in the preceding two weeks (Zhu et al., 2020). Afterward, the survived rats in the ISO-induced HF group were further randomly divided into ISO (n = 10) and ISO +TMZ group (n =10). Rats in ISO +TMZ group were given TMZ gavage at 10 mg/kg daily. In contrast, those in the ISO and control groups were given normal saline gavage at an identical volume for six consecutive weeks. Rats were euthanized after week eight, and blood samples were obtained. The rats’ hearts were collected and rinsed with precooled normal saline, then dissected and weighed. Meanwhile, we used one part of the tissues for measured protein concentration and subsequent experiments. The experiments included homogenates preparation and measurements of substrate contents. While the other part was fixed using 4% paraformaldehyde and paraffin-embedded for histology.

### Heart Weight Index (HWI) Assessment

To assess cardiac function, we excised and washed the rat hearts with precooled normal saline. After that, we carefully removed moisture on the surface of tissues with an absorbent paper for weighing. Afterwards, the heart weight index was calculated as follows: HWI (mg/g) = heart weight/body weight (n =10).

### Measurement of Serum B-Type Natriuretic Peptide (BNP) Levels

Fresh blood was collected in tubes and kept at room temperature until measurement within half an hour. Then we centrifuged the blood at 3,000 g for 10 min to separate serum. Then the samples were added in the buffer before permitted to equilibrate at room temperature for 30 min. Afterwards, we conducted all experimental operations according to kit instructions (Tianjin Anoric Biotechnology Co., Ltd, Tianjin, China) (n =6).

### Blood Pressure and Heart Rate

The rats were immobilized in the conscious state to ensure proper connectivity and maintained a suitable temperature and sense for operation. Systolic blood pressure, diastolic blood pressure, mean arterial pressure, and heart rate were measured using the tail-cuff method (BP–2010A, Softron Biotechnology Ltd., Beijing, China). All manipulations were replicated three times in a warm, and quiet environment, and calculated mean value (n = 6).

### Echocardiography

Before placed in the supine position for echocardiographic measurements, rats were anesthetized using 3% sodium pentobarbital solution (Tianjin Chemical Reagent Company, Tianjin, China) at 10.0 mg/kg. Next, the following indicators were measured: left ventricular end-systolic diameter (LVESD), left ventricular end-diastolic diameter (LVEDD), left ventricular end-diastolic volume (LVEDV) and left ventricular end-systolic volume (LVESV). Also, left ventricular fractional shortening (LVFS) and left ventricular ejection fraction (LVEF) were measured. Using an ultra-high resolution small animal ultrasound scanner Vevo^®^2100 (VisualSonics, Toronto, ON, Canada), transthoracic two-dimensionally guided M-mode echocardiography was performed (n = 6).

### Histopathological Analysis

Myocardial tissues of rats were fixed in 4% paraformaldehyde and dehydrated with segments embedded in paraffin. Then, we cut them into 5-μm thick sections for staining with Masson’s trichrome and hematoxylin-eosin solution. We observed the pathological changes in myocardial tissues using an optical microscope (DS-Ri2, Nikon, Japan) (n =5). Collagen volume fraction (CVF) was analyzed with Image J in multiple random visual fields of each sample, using the formula: CVF= collagen area/total observed area×100%.

### Measurement of ATP Contents

We used a certain amount of myocardial tissues for homogenate preparation with boiling double-distilled water (10% w/v). Immediately afterwards, the mixture was treated in a boiling water bath for 10 min, and then the homogenate was centrifuged at 3,500 g for 10 min. Finally, we collected the supernatant for the test. We carried out the specific operation according to the instructions (Nanjing Jiancheng Bioengineering Institute, Nanjing, China) (n =6).

### Measurement of Glucose, Free Fatty Acid, Lactic Acid, and Pyruvate Contents

A series of experiments were performed according to kit instructions (Solarbio, Beijing, China). Myocardial tissues were homogenized and centrifuged, and then the supernatant was collected for subsequent assays. A portion of supernatant was used to determine protein content using a bicinchoninic acid assay according to the manufacturer’s protocol (Solarbio, Beijing, China). At the same time, the others were used for absorbance measurement by a microplate reader (Epoch2, BioTek Instruments, Inc, America) (n = 6).

### Measurement of Ketone Body Contents

KB quantitative detection was calculated according to the manufacturer’s instructions (Tianjin Anoric Biotechnology Co., Ltd, Tianjin, China). Myocardial tissue was homogenized in lysis buffer containing a protease inhibitor and centrifuged at 3,000 g for 10 min, and then the supernatant was taken for subsequent experiments. Each well was added in samples and biotinylated antibody (1:1), with doubling doses of horseradish peroxidase-labeled antibody. Then the coated microwell plate was covered immediately and incubated at 37°C for 60 min. Next, the liquid mixture was discarded following by washing the plate five times with a washing buffer. After the last wash, the plate was left to air dry. Afterward, chromogenic reagent I and II were added to each well sequentially. Next, the mixture was incubated at room temperature for 20 min and reacted protected from light. Eventually, the termination buffer was added into each well, and the absorbance was read at 450 nm with a microplate reader (n = 6).

### TdT-Mediated dUTP Nick-End Labeling (TUNEL) Assays

Tunel staining using the TUNEL Apoptosis Detection Kit (Wanlei bio, Shenyang, China) was performed according to the manufacturer’s instructions. The paraffinized sections were dewaxed to water and rinsed with phosphate buffer saline (PBS) three times. Then, the tissue sections underwent heat-induced epitope retrieval in citrate buffer followed by a cooling-down period. Next, the paraffin sections were rinsed with PBS and added a tunel assay solution and then incubated at 37°C in the dark for 90 min. Sections were then rinsed with PBS and counterstained with 4’,6-diamidino-2-phenylindole (DAPI) for nuclear labeling. Finally, the anti-fluorescence quencher (Solarbio, Beijing, China) was used to seal the sections. The staining results were observed under a fluorescent microscope (DS-U3, Nikon, Japan), in which tunel-positive expression was recognized by green fluorescence, while DAPI-positive was blue. The apoptotic index’s final calculation was reached according to the following formula: apoptotic index=tunel positive cells/DAPI positive cells (n = 5).

### Protein Extraction and Western Blotting

Myocardial and hepatic tissue were homogenized in RIPA extraction buffer (Solarbio, Beijing, China) with phenylmethylsulfonyl fluoride, and phosphatase inhibitors (KeyGen Biotech. Inc, Nanjing, China)(100:1:1) in an ice bath. We centrifuged the homogenate at 14,000 g for five min. The obtained supernatant was then used for the detection of protein concentration before added loading buffer (3:1) and heated in a 100°C boiling water bath for 5 min. After cooling to room temperature, samples were centrifuged at 14,000 rpm for five min, and the supernatant was analyzed by sodium dodecyl sulfate-polyacrylamide gel electrophoresis (Solarbio, Beijing, China) and immunoblotting. Membrane and cytosolic proteins were extracted using the Membrane and Cytosol Protein Extraction Kit according to the manufacturer’s instructions. For immunoblotting, proteins were separated by SDS-PAGE and transferred to polyvinylidene difluoride membrane. The membrane was then blocked with a blocking buffer (Shanghai Biyuntian Bio-Technology Co., Ltd., Shanghai, China) 15 min at room temperature. After blocking, the membrane was incubated overnight at 4°C with primary antibodies, diluted by tris buffered saline tween. Next, the membrane was washed with tris buffered saline tween before incubated with HRP-tagged the second antibody and detected with luminol reagent (Engreen Biosystem Co, Ltd. Beijing, China). Finally, we analyzed the bands with Image J software (NIH, Bethesda, MD, USA) (n = 3).

### Statistical Analysis

All experimental data were expressed as mean ± standard deviation (SD), and statistical analysis was conducted using SPSS 21.0 statistical software (Lead Technologies, Chicago, USA). The differences between the groups were analyzed using a one-way analysis of variance (ANOVA), followed by Tukey’s multiple comparisons with a significance level of 0.05.

## Result

### TMZ Improves the General Condition and Cardiac Dysfunction of Rats in the ISO-Induced HF Model

**Figure 1:**
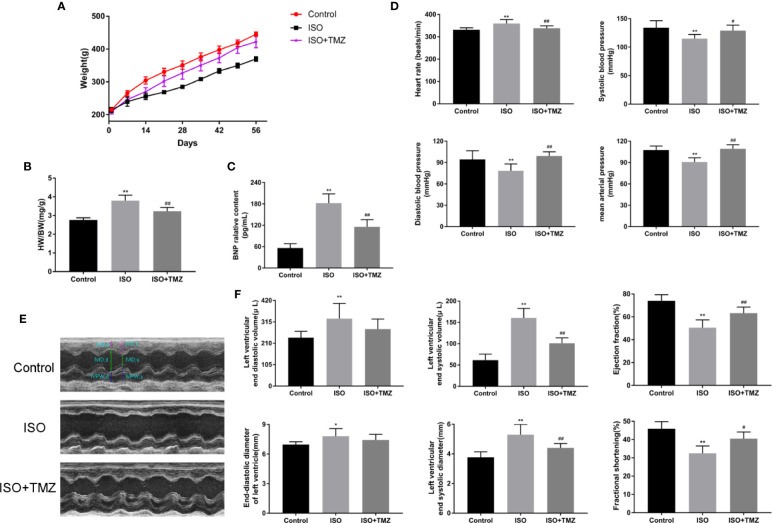
Trimetazidine improves the general condition and cardiac function of rats in the isoproterenol-induced HF model. **(A)** Bodyweight changes of rats in each group (n = 10). **(B)** Changes in rat heart weight index for different groups (n = 10). **(C)** B-type natriuretic peptide levels in sera were detected by enzyme linked immunosorbent assay (n = 6). **(D)** Heart rate, systolic blood pressure, diastolic blood pressure, mean arterial pressure of rats in each group (n =6). **(E)** Representative M-mode echocardiograms for different groups. **(F)** The histogram showed left ventricular end-diastolic volume, left ventricular end-systolic volume, and left ventricular ejection fraction. Left ventricular end-diastolic diameter, left ventricular end-systolic diameter and left ventricular fractional shortening (n = 6) were also shown. Data are represented as mean ± SD, ^#^P < 0.05, ^##^P < 0.01 compared with ISO group, *P < 0.05, **P < 0.01 compared with control group.

To evaluate the effect and mechanism of TMZ in improving heart function, we established an ISO-induced rat HF model. As shown in **Figure 1A**, the subcutaneous injection of ISO resulted in decreased bodyweight gain and increased HWI (**Figure 1B**) in rats. In contrast, TMZ treatment reduced the effect of ISO (**Figures 1A, B**). Furthermore, BNP levels were significantly elevated in the ISO group, and TMZ works oppositely (**Figure 1C**). Besides, there were significant changes in blood pressure and heart rate after subcutaneous injection of ISO in rats. The results indicated that TMZ intervention restrained the increase of heart rate and a drop in blood pressure (**Figure 1D**). We performed echocardiography to gain further insight into an alteration in myocardial morphology and function of rats in each group. The results showed that animals injected with ISO displayed ventricular remodeling at the end of week 8 (**Figure 1E**). Accompanied by the change, there was a decline in cardiac function, as assessed by increased LVESD, LVEDD, LVEDV, and LVESV. Meanwhile, LVEF and LVFS levels were decreased after week 8 (**Figure 1F**). However, TMZ attenuated these pathological changes. These results prove that TMZ attenuates the effect of ISO in inducing HF. Besides, TMZ improves the general condition and cardiac dysfunction of rats in the ISO-induced HF model.

### TMZ Ameliorates Pathological Changes in Myocardial Tissues of ISO-Induced Rats

**Figure 2:**
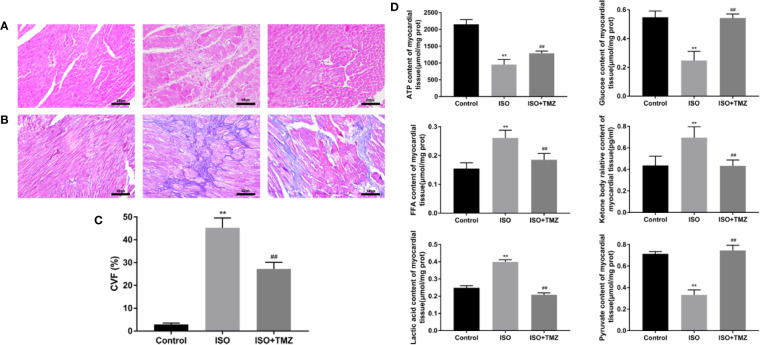
Trimetazidine ameliorates pathological changes in myocardial tissues of isoproterenol-induced rats. **(A)** Histopathological changes in myocardial tissues observed by hematoxylin-eosin staining (×200 magnification) (n = 5). **(B)** Masson’s Trichrome Staining of cardiac tissues (×200 magnification) (n = 5). **(C)** The Collagen Volume Fraction of myocardial tissues was calculated as quantification (n= 5). **(D)** Myocardium was homogenized, and the content of ATP, glucose, free fatty acid, ketone body, lactic acid, and pyruvate were detected following the instructions (n = 6). Data were represented as mean ± SD, ^##^P < 0.01 compared with ISO group, **P < 0.01 compared with control group.

We conducted hematoxylin–eosin staining and Masson’s trichrome staining for pathological assessment to evaluate the histopathology changes in ISO-induced rats’ myocardial tissues. Besides, we measure the content of metabolic substrates. In comparison with the control group, animals treated with ISO displayed myocardial fibers disarray and myocyte hypertrophy. However, these changes were remarkably prevented by TMZ (**Figure 2A**). Similar results were seen with Masson’s Trichrome Staining that heart tissues demonstrated myocardial cell swelling, and the myocardial fibers were in a disorganized array. While TMZ attenuated these pathological changes (**Figure 2B**). Besides, ISO caused a marked increase in CVF compared with the control group. Whereas treated with TMZ can remarkably prevent the variation (**Figure 2C**). As shown in **Figure 2D**, ISO stimulation resulted in an increased accumulation of free fatty acid, ketone bodies, and lactic acid. However, there was a decrease in levels of ATP, glucose, and pyruvate. In contrast, treated with TMZ can remarkably prevent the accumulation of the acidic metabolite and improve insufficiencies of ATP, glucose, and pyruvate. These results indicate that TMZ attenuated the histopathology alterations and improved the utilization of cardiac metabolism substrate in ISO-induced HF rats.

### TMZ Reduces Cell Apoptosis in Myocardial Tissues and Increases the Ketogenesis-Related Protein Expression Level of Hepatic Tissue in ISO-Induced HF Rats

**Figure 3:**
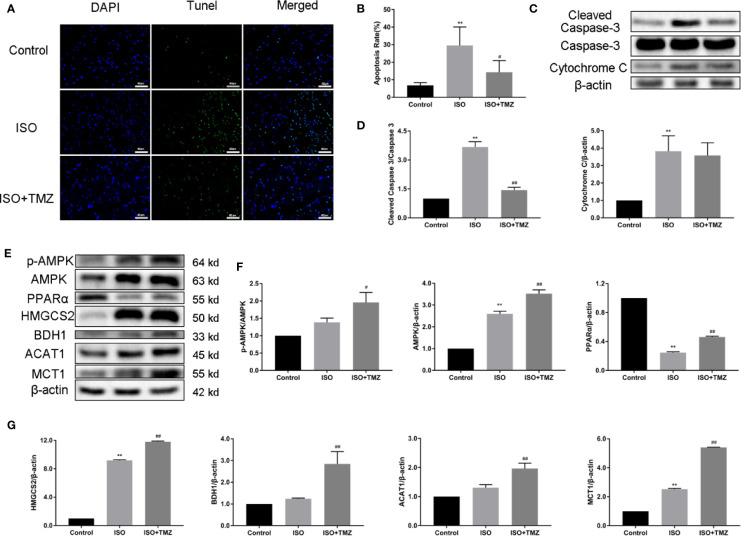
Trimetazidine reduces cell apoptosis of myocardial tissues and increases the ketogenesis-related protein expression level of hepatic tissue in heart failure rats. **(A)** Representative images of DNA fragmentation as tested by tunel assay and DAPI staining (×400 magnification) for different groups. **(B)** The number of apoptotic cells was calculated by taking five views in each group (n = 5). **(C)** Western blot was used to detect the expression levels of cleaved caspase 3, caspase 3 and cytochrome C. **(D)** The density of the immunoreactive bands was analyzed by Image J software. **(E)** Western blot was used to evaluate ketogenesis-related protein expression. AMPK, p-AMPK, PPARα, HMGCS2, BDH1, MCT1 and ACAT1 were included. **(F, G)** The histogram indicated the specific relevant quantitative results (n = 3). Data are represented as means ± SD, ^#^P < 0.05, ^##^P < 0.01 compared with ISO group, **P < 0.01 compared with control group. AMPK, adenosine monophosphate-activated protein kinase; p-AMPK, phospho-AMPK; PPARα, peroxisome proliferator activated receptorα; HMGCS2, 3-hydroxy-3-methylglutaryl-CoA synthase 2; BDH1, β-hydroxybutyrate dehydrogenase 1; MCT1, monocarboxylate transporter 1; ACAT1, acetoacetyl-CoA thiolase 1.

To explore the cell apoptosis in myocardial tissues, we performed Tunel staining and western blot. ISO can induce cell apoptosis in myocardial tissues was proved, whereas the increased ratio of cleaved caspase 3/caspase 3 can be reduced by TMZ intervention. After the TMZ intervention, cytochrome C level was declined, but there was no statistical difference. Furthermore, to determine whether TMZ acts on ketogenesis, we evaluated the protein expression related to ketogenesis in hepatic tissue. As shown in **Figures 3E–G**, ISO, markedly down-regulated PPARα expressions, and upregulated AMPK, HMGCS2, and MCT1. However, TMZ intervention was further increased the protein expression of AMPK, HMGCS2, and MCT1. More than that, PPARα, BDH1, ACAT1 expression, and the ratio of p-AMPK/AMPK were significantly improved. These results suggest that TMZ can reduce cell apoptosis in myocardial tissues to a certain extent. Besides this, TMZ can promote protein expression related to ketogenesis *via* the activated AMPK/PPARα signaling pathway.

### TMZ Promotes the Protein Expression Related to Myocardial Substrate Uptake and Utilization With Activation of the AMPK/PPARα Signaling Pathway in HF Rats

**Figure 4:**
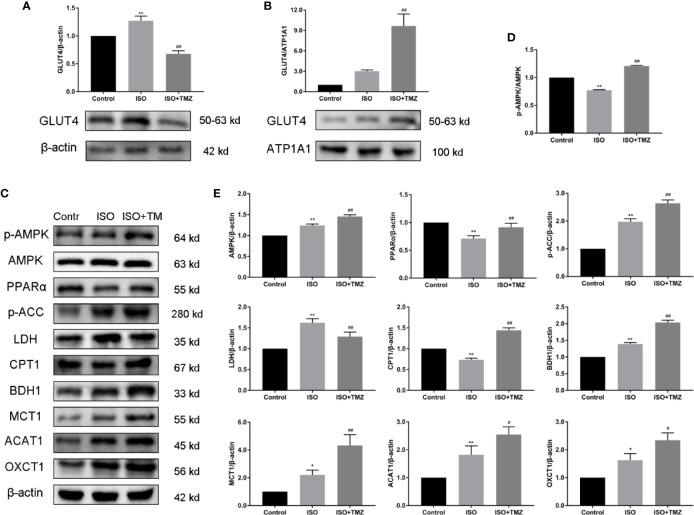
Trimetazidine treatment promotes the protein expression related to myocardial substrate uptake and utilization in myocardial tissues of isoproterenol-induced heart failure rats. **(A)** Cytosolic protein was extracted and expression levels of cellular localization of GLUT4 were determined using Western blot. β-actin was choice as an internal control. **(B)** Membrane protein was extracted and expression levels of cellular localization of GLUT4 were determined using Western blot. ATP1A1 was chosen as an internal control. **(C)** Western blot was used to evaluate metabolism-related protein expression. AMPK, p-AMPK, PPARα, p-ACC, LDH, CPT1, BDH1, MCT1, ACAT1, and OXCT1 were included. **(D)** Immunoblotting analysis was conducted to detect the ratio of p-AMPK/AMPK protein expression within myocardial tissues (n = 3). **(E)** The histogram indicated the specific relevant quantitative results (n = 3). Data are represented as means ± SD, ^#^P < 0.05, ^##^P < 0.01 compared with ISO group, *P < 0.05, **P < 0.01 compared with control group. p-AMPK, phospho-AMPK; AMPK, adenosine monophosphate-activated protein kinase; PPARα, peroxisome proliferator activated receptorα; p-ACC, phospho-acetyl-CoA carboxylase; LDH, lactate dehydrogenase;CPT1, carnitine palmitoyltransferase 1; BDH1,β-hydroxybutyrate dehydrogenase 1; MCT1, monocarboxylate transporter 1; ACAT1, acetoacetyl-CoA thiolase 1; OXCT1(SCOT1), succinyl-CoA:3-ketoacid-CoA transferase1.

To investigate the mechanism of TMZ effect on energy metabolism of myocardial tissue in ISO-induced HF rat, we evaluated protein expressions using western blot. These protein expressions related to myocardial substrate uptake and utilization and activation of AMPK/PPARα signaling pathway. As shown in **Figures 4A, B**, TMZ significantly promoted translocation of GLUT4 from cytosolic to membrane. As illustrated in **Figures 4C–E**, ISO PPARα, CPT1 protein expression were down-regulated, and AMPK, p-ACC, LDH, BDH1, MCT1, ACAT1, and OXCT1 protein expression were upregulated. At the same time, the ratio of p-AMPK/AMPK protein expression was decreased. However, treated with TMZ can remarkably increase the phosphorylation of AMPK, which is characterized by an increase in the ratio of p-AMPK/AMPK protein expression. Besides, TMZ has a reversal of depletion in PPARα, GLUT4, CPT1 protein levels. Furthermore, TMZ intervention further promoted p-ACC, LDH, BDH1, MCT1, ACAT1, and OXCT1 protein expression. These results suggest that TMZ could activate the AMPK/PPARα signaling pathway and promote protein expression related to myocardial substrate uptake and utilization.

## Discussion

Along with TMZ preconditioning in a single-dose, the plasma levels of adenosine was increased, as one study showed (Liu et al., 2016). Many studies have concluded that only treated HF with TMZ can partly improve indices of serum (Zhang et al., 2012). Even with the addition of calcium channel blockers, symptoms in patients with stable angina can only be partially improved (Marzilli et al., 2019). Recent analyses have identified that TMZ demonstrates excellent results in HF patients, including decreasing the prevalence and incidence of cardiovascular events (Zhang et al., 2012; Grajek and Michalak, 2015). However, the exact mechanism of how TMZ develops its full potential in improving cardiac energy metabolism in HF is not well understood and needs further study. Therefore, we have investigated the mechanism of trimetazidine in the ISO-induced heart failure rat model. Trimetazidine can prevent or reverse the progression of cardiac dysfunction and metabolic remodeling in our experiments. The remarkable performance of trimetazidine in HF may be closely associated with AMPK and PPARα, whose expression increases obviously in our study.

ISO is a beta-adrenergic receptor agonist that could inhibit both β1 and β2. ISO is usually used to induce pathologic myocardial diseases experimentally (Fan et al., 2019). ISO can bring about myocardial ischemia, further resulting in a particular form of metabolic and ultrastructural variations that induce irreversible injury. The alterations cause different types of cardiomyocyte death, referred to as apoptosis and necrosis. Besides, ISO is adequate to induce myocardial hypertrophy, inflammation, and fibrosis (Benjamin et al., 1989; Zhang et al., 2020). These changes may cause an increase in the ratio of heart weight to body weight (Freund et al., 2005). The same results were found in this study. These changes are shown and ultimately predispose the heart to diastolic and systolic dysfunction with myocardial metabolic remodeling (van Bilsen et al., 2004). The changes in rat associated with ISO-induced HF are similar to the pathological changes of myocardial tissue in the process of human heart failure. Hence ISO-induced HF in rats is an extensively applied model in experimental medical research (Fan et al., 2019). The heart failure model in our study was induced by subcutaneous injection with ISO. In this study, cardiac function parameters measured by echocardiography, including LVEF and LVFS, were significantly reduced in the ISO intervention group. These changes verified the HF model in rats. Besides, heart rate and blood pressure were decreased while the plasma levels of BNP were significantly increased after ISO intervention (Wang et al., 2018; Fan et al., 2019). These results concur with previous studies. Nevertheless, trimetazidine performed well in improving biochemical parameters, blood pressure, and pathological changes. These performances provided strong evidence that trimetazidine has enormous potential in ameliorating cardiac function and slows HF progression. In this study, We verified the benefits of trimetazidine proved in a clinical trial.

Previous research had shown that heart failure results in an increase in AMPK activity in mice and humans (Quan et al., 2017; Zhang et al., 2017). Zhuo et al. indicated that activation of AMPK could inhibit BNP elevation and cardiomyocyte apoptosis in a rat model of heart failure induced by ISO (Zhuo et al., 2013). Available research considered that the inhibition of c-Jun N-terminal kinase and nuclear factor kappa-B is correlated with AMPK activation. The restraint may lead to attenuation in inflammation and a decrease in cell death (Chen et al., 2018; Gu et al., 2020). Besides, some studies showed that the activation state for AMPK often leads to inhibiting extracellular signal-regulated kinase. This effect could improve cardiac fibrosis (Du et al., 2008). It is considered when the myocardial injury occurs, AMPK in hearts will be activated as a compensatory reaction. As the disease progresses, compensatory responses of AMPK activation were not strong enough to satisfy various demands, and then the pathologic changes arise. Fortunately, TMZ could relieve these pathologic changes by further activation of AMPK in this study. Besides, AMPK-mediated transportation of GLUT4 from the cytosol to the membrane increases glucose uptake (Li et al., 2019). This increase often acts as an adaptive and protective response of the heart. AMPK can also increase fatty acid uptake and oxidation by increasing the activity of CPT-1, which was conducted by inhibiting acetyl-CoA carboxylase. These are also confirmed in our study. CPT1 serves as a limiting step for long-chain fatty acid accessing to the mitochondria for β-oxidation, which is vital to ATP production (Dyck and Lopaschuk, 2006). Moreover, AMPK plays a very notable reverse role in regulating the mammalian target of rapamycin 1. The inhibition of mTORC1 is mainly started by AMPK. Further, the inhibition state of mTORC1 is necessary for the activation of ketogenesis in the liver (Grabacka et al., 2016). So we have reason to believe that AMPK is closely related to ketone body’s metabolism. It is worth mentioning that TMZ can partially inhibit fatty acid oxidation, decreasing the nicotinamide adenine dinucleotide reduced/oxidized ratio. The decrease can lead to the enhancement of pyruvate dehydrogenase activity and efficiency increase in glucose and pyruvate oxidation (Li et al., 2019). Studies showed that TMZ could enhance the activity of AMPK through its effects on the ATP level in cardiomyocytes (Liu et al., 2016). In this study, AMPK carries out the function of regulating substrate metabolism by itself phosphorylation. Early in the study, rats treated with iso displayed compensatory changes by activating AMPK, which may have a good effect on myocardial metabolism. As the disease progresses, myocardium showed obvious decompensation, with energy metabolism disorder including reduction of ATP production and acid metabolite accumulation. However, it is worth noting that TMZ can notably activate AMPK and then increase protein expression involved in GLUT4 and CPT1. Along with the activation of AMPK, proteins expression levels of ketone body metabolism raised. Therefore, as mentioned in the present study, we think TMZ treatment can improve the metabolic status and reduce progression to heart failure *via* shifting substrate metabolism. The function most likely has a close relationship with the AMPK pathway.

KB, considered efficient fuel, is meaningful in heart failure (Aubert et al., 2016; Bedi et al., 2016). Ketone bodies are usually formed as an intermediate product of fatty acid oxidation, including β-hydroxybutyrate, acetoacetate, and acetone (Puchalska and Crawford, 2017). The production of ketone bodies mainly occurs in the mitochondria of hepatocytes, but the liver is the only tissue without the ability of ketolysis. Ketone bodies (β-hydroxybutyrate and acetoacetate) are generally transported by monocarboxylate transporter 1 (MCT1) to tissues other than hepatocytes who express OXCT genes (Halestrap and Wilson, 2012; Puchalska and Crawford, 2017). Ketogenesis and ketolysis are regulated by multiple factors, in which PPARα is a critical aspect. The previous report showed inconsistent expression of PPARα in cardiovascular diseases (Kodde et al., 2007). Studies show that PPARα is necessary for the initiation and transcription of 3-hydroxy-3-methylglutaryl-CoA synthase. At the same time, HMGCS2 acted as a speed-limiting enzyme for the generation of ketone bodies (Grabacka et al., 2016). Research showed that the expression of ketolysis-related proteins, including BDH1 and OXCT1, was increased in patients with heart failure (Bedi et al., 2016). Along with growing ketone body oxidation of myocardial tissue, the pathological process of HF may be delayed. In this study, we observed that the expression of PPARα was increased, secondary to AMPK activation, with TMZ therapy. The protein expressions referred to ketone body production and utilization were upregulated in ISO-induced HF rats. By increasing the ketone body utilization, cardiac function was improved with an enhancement of ATP production.

The failing heart could improve ATP production by reducing cardiac fatty acid utilization and shifting toward a greater reliance on glycolysis and ketone body oxidation (Karwi et al., 2018). In this state, it seems that glucose and pyruvate’s content should be increased following energy demand to increase ATP production, which can be realized by increasing glucose intake. The high expression of GLUT4 is advantageous to glucose intake. Nevertheless, these conditions could not be achieved in the failing heart, promoting the progress of HF. Alike, due to the decreased demand, the failing heart should decrease the uptake to reduce the content of FFA. Although the expression of CPT1 was reduced, the failing heart still showed low utilization of FFA, so the content of FFA was still high, which leads to further development of HF. Besides, the highly expressed MCT1 increased KB intake, but the failing heart was only able to utilize a limited KB. Therefore, the KB content was high, and the failing heart still could not meet the energy demand. Besides, When the oxygen supply is insufficient in the failing heart, lactic acid is produced by anaerobic glycolysis, which transforms pyruvate into lactic acid by the lactate dehydrogenase enzyme. However, excessive lactic acid generation is not able to be fully utilized to meet cardiac energy demand. So, the lactic acid content was increased while the pyruvate content decreased, leading to a further energy imbalance and eventually resulting in the decline of cardiac function. However, following the TMZ intervention, the glucose and pyruvate’s content was increased to provide sufficient metabolic substrate, which may benefit from the highly expressed GLUT4. Meanwhile, the accumulation of lactic acid is improved. The CPT1 was highly expressed, and the FFA content was decreased instead in the group of ISO+TMZ, which is most likely due to increased conversion and utilization of FA. Indeed, the KB, which was derived from fatty acids, was in a hypermetabolic state. So, MCT1 and other KB metabolism-related protein expressions were further increased in the group of ISO+TMZ. This series of changes showed that TMZ could increase uptake of glucose, FFA, and KB and, at the same time, increase the glucose and KB utilization and FA conversion and utilization. Eventually, the overall effectiveness of metabolism was improved to meet the cardiac energy demand, and cardiac function was improved to delay the process of HF.

The limitation of the present study is that metabolomics was not conducted. Energy metabolism in HF rats and the effect of TMZ on myocardial should be further unambiguously confirmed. The confirmation should be based on high-throughput sequencing analysis through metabolomics. Besides, the link between TMZ and AMPK/PPARα has not been verified by *in vitro* experiments. We will address these limitations in future studies.

Our study results show that myocardial structure damage and cardiac dysfunction in ISO-induced HF rats are related to metabolic remodeling. Myocardial substrate uptake and utilization act as the initiations of energy metabolism. TMZ can promote protein expression related to substrate uptake and utilization *via* upregulating AMPK and PPARα expression. The regulation is closely related to the metabolism of ketone bodies. Moreover, The regulation ameliorates the reduction of ATP production and a decline in cardiac function. This study provides a reference for further studies.

## Data Availability Statement

The raw data supporting the conclusions of this article will be made available by the authors, without undue reservation, to any qualified researcher.

## Ethics Statement

The animal study was reviewed and approved by the Animal Care and Use Committee of the Tianjin Union Medical Center.

## Author Contributions

HL conceived and designed the study; collected, analyzed and interpreted the data; and assisted in writing the manuscript. ZM conceived and designed the study and collected and presented the data. YZ and CL were involved in animal experiments. PY and FZ assessed western blotting analyses. LW analyzed and interpreted the data. QL and XQ conceived and designed the study, wrote the manuscript, and gave final approval of the manuscript.

## Funding

This research was supported by the Tianjin Science and Technology Planning Project (Grant No. 16ZXMJSY00060), Cooperation Project of Beijing, Tianjin and Hebei (grant no. 19JCZDJC63900) and Foundation of Tianjin Union Medical Center (grant nos. 2019YJ016 and 2019YJZD001) and the 2018 Annual Graduate Students Innovation Fund (Grant No. ZXYCXLX201811).

## Conflict of Interest

The authors declare that the research was conducted in the absence of any commercial or financial relationships that could be construed as a potential conflict of interest.

## Abbreviations

HF, Heart failure; FFA, free fatty acids; ATP, adenosine triphosphate; KB, ketone body; TMZ, Trimetazidine; ISO, isoproterenol; AMPK, adenosine monophosphate-activated protein kinase; PPARα, peroxisome proliferator activated receptorα; LDH, lactate dehydrogenase; GLUT4, glucose transporter 4; p-ACC, phospho-acetyl-CoA carboxylas; HMGCS2, 3-hydroxy-3-methylglutaryl-CoA2; MCT1, monocarboxylate transporter 1; BDH1,β-hydroxybutyrate dehydrogenase 1; p-AMPK, phospho-AMPK; CPT1, carnitine palmitoyltransferase 1; ACAT1, acetoacetyl-CoA thiolase 1; OXCT1(SCOT1), succinyl-CoA:3-ketoacid-CoA transferase1; BNP, B-type Natriuretic Peptide; CVF, Collagen volume fraction; TUNEL, TdT-mediated dUTP Nick-End Labeling; PBS, phosphate buffer saline; DAPI, 4’,6-diamidino-2-phenylindole.

## References

[B1] Aubert G. Martin O. J. Horton J. L. Lai L. Vega R. B. Leone T. C. (2016). The Failing Heart Relies on Ketone Bodies as a Fuel. Circulation 133 (8), 698–705. 10.1161/circulationaha.115.017355 26819376 PMC4766035

[B2] Bedi K. C. Snyder N. W. Brandimarto J. Aziz M. Mesaros C. Worth A. J. (2016). Evidence for Intramyocardial Disruption of Lipid Metabolism and Increased Myocardial Ketone Utilization in Advanced Human Heart Failure. Circulation 133 (8), 706–716. 10.1161/circulationaha.115.017545 26819374 PMC4779339

[B3] Benjamin I. J. Jalil J. E. Tan L. B. Cho K. Weber K. T. Clark W. A. (1989). Isoproterenol-induced myocardial fibrosis in relation to myocyte necrosis. Circ. Res. 65 (3), 657–670. 10.1161/01.res.65.3.657 2527639

[B4] Benjamin E. J. Blaha M. J. Chiuve S. E. Cushman M. Das S. R. Deo R. (2017). Heart Disease and Stroke Statistics-2017 Update: A Report From the American Heart Association. Circulation 135 (10), e146–e603. 10.1161/cir.0000000000000485 28122885 PMC5408160

[B5] Birkenfeld A. L. Adams F. Schroeder C. Engeli S. Jordan J. (2011). Metabolic actions could confound advantageous effects of combined angiotensin II receptor and neprilysin inhibition. Hypertension (Dallas Tex 1979) 57 (2), e4–e5. 10.1161/hypertensionaha.110.165159 21149824

[B6] Chen X. Li X. Zhang W. He J. Xu B. Lei B. (2018). Activation of AMPK inhibits inflammatory response during hypoxia and reoxygenation through modulating JNK-mediated NF-κB pathway. Metabolism: Clin. Exp. 83, 256–270. 10.1016/j.metabol.2018.03.004 PMC5960613 29526538

[B7] Chen L. Song J. Hu S. (2019). Metabolic remodeling of substrate utilization during heart failure progression. Heart failure Rev. 24 (1), 143–154. 10.1007/s10741-018-9713-0 29789980

[B8] Du J. Guan T. Zhang H. Xia Y. Liu F. Zhang Y. (2008). and AMPK in the growth and proliferation of cardiac fibroblasts. Biochem. Biophys. Res. Commun. 368 (2), 402–407. 10.1016/j.bbrc.2008.01.099 18243130

[B9] Dyck J. R. Lopaschuk G. D. (2006). AMPK alterations in cardiac physiology and pathology: enemy or ally? J. Physiol. 574, 95–112. 10.1113/jphysiol.2006.109389 16690706 PMC1817803

[B10] Fan C. Tang X. Ye M. Zhu G. Dai Y. Yao Z. (2019). viaQi-Li-Qiang-Xin Alleviates Isoproterenol-Induced Myocardial Injury by Inhibiting Excessive Autophagy Activating AKT/mTOR Pathway. Front. Pharmacol. 10, 1329. 10.3389/fphar.2019.01329 31780944 PMC6861302

[B11] Freund C. Schmidt-Ullrich R. Baurand A. Dunger S. Schneider W. Loser P. (2005). Requirement of nuclear factor-kappaB in angiotensin II- and isoproterenol-induced cardiac hypertrophy in vivo. Circulation 111 (18), 2319–2325. 10.1161/01.Cir.0000164237.58200.5a 15870116

[B12] Grabacka M. Pierzchalska M. Dean M. Reiss K. (2016). Regulation of Ketone Body Metabolism and the Role of PPARα. Int. J. Mol. Sci. 17 (12), 2093. 10.3390/ijms17122093 PMC5187893 27983603

[B13] Grajek S. Michalak M. (2015). The effect of trimetazidine added to pharmacological treatment on all-cause mortality in patients with systolic heart failure. Cardiology 131 (1), 22–29. 10.1159/000375288 25832112

[B14] Gu X. Li Y. Chen K. Wang X. Wang Z. Lian H. (2020). Exosomes derived from umbilical cord mesenchymal stem cells alleviate viral myocarditis through activating AMPK/mTOR-mediated autophagy flux pathway. J. Cell. Mol. Med. 24 (13), 7515–7530. 10.1111/jcmm.15378 PMC7339183 32424968

[B15] Halestrap A. P. Wilson M. C. (2012). The monocarboxylate transporter family–role and regulation. IUBMB Life 64 (2), 109–119. 10.1002/iub.572 22162139

[B16] Karwi Q. G. Uddin G. M. Ho K. L. Lopaschuk G. D. (2018). Loss of Metabolic Flexibility in the Failing Heart. Front. Cardiovasc. Med. 5, 68. 10.3389/fcvm.2018.00068 29928647 PMC5997788

[B17] Kodde I. F. van der Stok J. Smolenski R. T. de Jong J. W. (2007). Metabolic and genetic regulation of cardiac energy substrate preference. Comp. Biochem. Physiol. Part A Mol. Integr. Physiol. 146 (1), 26–39. 10.1016/j.cbpa.2006.09.014 17081788

[B18] Kolwicz S. C. Airhart S. Tian R. (2016). Ketones Step to the Plate: A Game Changer for Metabolic Remodeling in Heart Failure? Circulation 133 (8), 689–691. 10.1161/circulationaha.116.021230 26819375 PMC4826559

[B19] Li X. Liu J. Lu Q. Ren D. Sun X. Rousselle T. (2019). AMPK: a therapeutic target of heart failure-not only metabolism regulation. Bioscience Rep. 39 (1). 10.1042/bsr20181767 PMC6328861 30514824

[B20] Liu Z. Chen J. M. Huang H. Kuznicki M. Zheng S. Sun W. (2016). The protective effect of trimetazidine on myocardial ischemia/reperfusion injury through activating AMPK and ERK signaling pathway. Metabolism: Clin. Exp. 65 (3), 122–130. 10.1016/j.metabol.2015.10.022 PMC4967934 26892523

[B21] Lopaschuk G. D. Barr R. Thomas P. D. Dyck J. R. (2003). Beneficial effects of trimetazidine in ex vivo working ischemic hearts are due to a stimulation of glucose oxidation secondary to inhibition of long-chain 3-ketoacyl coenzyme a thiolase. Circ. Res. 93 (3), e33–e37. 10.1161/01.Res.0000086964.07404.A5 12869392

[B22] Marzilli M. Vinereanu D. Lopaschuk G. Chen Y. Dalal J. J. Danchin N. (2019). Trimetazidine in cardiovascular medicine. Int. J. Cardiol. 293, 39–44. 10.1016/j.ijcard.2019.05.063 31178223

[B23] Nakamura M. Sadoshima J. (2019). Ketone body can be a fuel substrate for failing heart. Cardiovasc. Res. 115 (11), 1567–1569. 10.1093/cvr/cvz104 30989167 PMC6704386

[B24] Ponikowski P. Voors A. A. Anker S. D. Bueno H. Cleland J. G. Coats A. J. (2016). ESC Guidelines for the diagnosis and treatment of acute and chronic heart failure: The Task Force for the diagnosis and treatment of acute and chronic heart failure of the European Society of Cardiology (ESC). Developed with the special contribution of the Heart Failure Association (HFA) of the ESC. Eur. J. Heart failure 18 (8), 891–975. 10.1002/ejhf.592 27207191

[B25] Puchalska P. Crawford P. A. (2017). Multi-dimensional Roles of Ketone Bodies in Fuel Metabolism, Signaling, and Therapeutics. Cell Metab. 25 (2), 262–284. 10.1016/j.cmet.2016.12.022 28178565 PMC5313038

[B26] Quan N. Sun W. Wang L. Chen X. Bogan J. S. Zhou X. (2017). Sestrin2 prevents age-related intolerance to ischemia and reperfusion injury by modulating substrate metabolism. FASEB J. Off. Publ. Fed. Am. Societies Exp. Biol. 31 (9), 4153–4167. 10.1096/fj.201700063R PMC5572689 28592638

[B27] Rosano G. M. Vitale C. (2018). Metabolic Modulation of Cardiac Metabolism in Heart Failure. Cardiac failure Rev. 4 (2), 99–103. 10.15420/cfr.2018.18.2 PMC6125709 30206484

[B28] van Bilsen M. Smeets P. J. Gilde A. J. (2004). GJ van der Vusse. Metabolic remodelling of the failing heart: the cardiac burn-out syndrome? Cardiovasc. Res. 61 (2), 218–226. 10.1016/j.cardiores.2003.11.014 14736538

[B29] Wang P. Xu L. Sun. Energy Remodeling A. (2016). Mitochondrial Disorder and Heart Failure. Curr. Pharm. design 22 (31), 4823–4829. 10.2174/1381612822666160708224330 27396599

[B30] Wang J. Wu M. L. Cao S. P. Cai H. Zhao Z. M. Song Y. H. (2018). Cycloastragenol ameliorates experimental heart damage in rats by promoting myocardial autophagy via inhibition of AKT1-RPS6KB1 signaling. Biomedicine pharmacotherapy = Biomedecine pharmacotherapie 107, 1074–1081. 10.1016/j.biopha.2018.08.016 30257319

[B31] Zhang L. Lu Y. Jiang H. Zhang L. Sun A. Zou Y. (2012). Additional use of trimetazidine in patients with chronic heart failure: a meta-analysis. J. Am. Coll. Cardiol. 59 (10), 913–922. 10.1016/j.jacc.2011.11.027 22381427

[B32] Zhang J. Zhao P. Quan N. Wang L. Chen X. Cates C. (2017). The endotoxemia cardiac dysfunction is attenuated by AMPK/mTOR signaling pathway regulating autophagy. Biochem. Biophys. Res. Commun. 492 (3), 520–527. 10.1016/j.bbrc.2017.08.034 28807827 PMC5593793

[B33] Zhang N. Zhang Y. Qian H. Wu S. Cao L. Sun Y. (2020). Selective targeting of ubiquitination and degradation of PARP1 by E3 ubiquitin ligase WWP2 regulates isoproterenol-induced cardiac remodeling. Cell Death Differentiation. 10.1038/s41418-020-0523-2 PMC7429876 32139900

[B34] Zhu Z. Li H. Chen W. Cui Y. Huang A. Qi X. (2020). Perindopril Improves Cardiac Function by Enhancing the Expression of SIRT3 and PGC-1α in a Rat Model of Isoproterenol-Induced Cardiomyopathy. Front. Pharmacol. 11, 94. 10.3389/fphar.2020.00094 32153406 PMC7046591

[B35] Zhuo X. Z. Wu Y. Ni Y. J. Liu J. H. Gong M. Wang X. H. (2013). Isoproterenol instigates cardiomyocyte apoptosis and heart failure via AMPK inactivation-mediated endoplasmic reticulum stress. Apoptosis an Int. J. programmed Cell Death 18 (7), 800–810. 10.1007/s10495-013-0843-5 23620435

